# Predicting and Empowering Health for Generation Z by Comparing Health Information Seeking and Digital Health Literacy: Cross-Sectional Questionnaire Study

**DOI:** 10.2196/47595

**Published:** 2023-10-30

**Authors:** Wen Jiao, Angela Chang, Mary Ho, Qianfeng Lu, Matthew Tingchi Liu, Peter Johannes Schulz

**Affiliations:** 1 School of Communication Soochow University Suzhou China; 2 Department of Communication, Faculty of Social Sciences University of Macau Macao China; 3 Institute of Communication and Health University of Lugano Lugano Switzerland; 4 Faculty of Communication, Culture and Society University of Lugano Lugano Switzerland; 5 Faculty of Business and Administration University of Macau Macao China; 6 Department of Communications and Media Ewha Womans University Seoul Republic of Korea

**Keywords:** health information seeking, digital health literacy, health empowerment, Generation Z, digitally savvy

## Abstract

**Background:**

Generation Z (born 1995-2010) members are digital residents who use technology and the internet more frequently than any previous generation to learn about their health. They are increasingly moving away from conventional methods of seeking health information as technology advances quickly and becomes more widely available, resulting in a more digitalized health care system. Similar to all groups, Generation Z has specific health care requirements and preferences, and their use of technology influences how they look for health information. However, they have often been overlooked in scholarly research.

**Objective:**

First, we aimed to identify the information-seeking preferences of older individuals and Generation Z (those between the ages of 18 and 26 years); second, we aimed to predict the effects of digital health literacy and health empowerment in both groups. We also aimed to identify factors that impact how both groups engage in digital health and remain in control of their own health.

**Methods:**

The Health Information National Trends Survey was adopted for further use in 2022. We analyzed 1862 valid data points by conducting a survey among Chinese respondents to address the research gap. A descriptive analysis, 2-tailed *t* test, and multiple linear regression were applied to the results.

**Results:**

When compared with previous generations, Generation Z respondents (995/1862, 53.44%) were more likely to use the internet to find out about health-related topics, whereas earlier generations relied more on traditional media and interpersonal contact. Web-based information-seeking behavior is predicted by digital health literacy (Generation Z: β=.192, *P*<.001; older population: β=.337, *P*<.001). While this was happening, only seeking health information from physicians positively predicted health empowerment (Generation Z: β=.070, *P*=.002; older population: β=.089, *P*<.001). Despite more frequent use of the internet to learn about their health, Generation Z showed lower levels of health empowerment and less desire to look for health information, overall.

**Conclusions:**

This study examined and compared the health information–seeking behaviors of Generation Z and older individuals to improve their digital health literacy and health empowerment. The 2 groups demonstrated distinct preferences regarding their choice of information sources. Health empowerment and digital health literacy were both significantly related to information-seeking behaviors.

## Introduction

### Overview

The term “Generation Z” refers to anyone born after 1995 who is known for being digitally savvy and consuming information through digital media [1]. Generation Z members, who are between the ages of 18 and 26 years and have grown up with access to digital technology, are more likely to look for web-based health-related material and use social media to interact with others about such issues [2].

Generation Z members are different from older generations because they are the first consumers to have grown up wholly in the digital era. Early research indicates that younger participants tend to have stronger computer skills and are more likely to report accessing the internet for health information [2]. In comparison, older individuals prefer to access traditional sources such as libraries and paper-based materials when seeking health information [3]. The emergence of Generation Z marks a new era in digital technology and information accessibility, as this generation has grown up in a world where information is just a click away and digital devices are an integral part of their daily lives.

### Background

Digital health literacy, being able to access health information, and feeling empowered in terms of one’s health are strongly correlated [4,5]. It is critical to comprehend the digital health literacy skills of Generation Z and how they compare to those of earlier generations, as information-seeking behavior continues to move toward web-based platforms. Particularly for Generation Z and older Chinese generations, these aspects could vary.

Individuals’ health behaviors are affected by the health information they seek out. An individual seeking health information is more likely to adopt healthier habits such as regular exercise, a balanced diet, and medication compliance [6,7]. Previous research is also clear on the effects of digital health literacy and empowerment on people’s health [8,9]. Meanwhile, a patient’s capacity to handle their care increases their feelings of being empowered, which will impact their health outcomes [10].

Numerous studies have examined the connection among health information–seeking behavior (HISB), digital health literacy, and health empowerment [11-13]. These studies indicated that increased digital health literacy and empowerment were linked with more frequent seeking of health information. Furthermore, web-based information seeking showed a positive relationship with health literacy and empowerment and was linked to improved health outcomes [8,9]. The following discussion of HISB, digital health literacy, and health empowerment thus includes parts connecting these relationships between Generation Z members and others.

Rarely does the present research provide a theoretical explanation of this new digital media culture. For instance, to conceptualize the desires, motivations, and media habits of Generation Z, Russians between the ages of 10 and 19 years were interviewed [14]. It was concluded that there is a direct connection between the rising use of social media and how it affects the environment in light of Generation Z’s ambitions for media consumption. Because social media incorporates components of both the media and the social system, it alters an organization’s ability to match audience expectations.

An indicator of digital inclusion specifically for the Russian media environment was created by Gladkova et al [15] in 2022. Russians use more web-based access devices than Yakuts (an ethnic minority), including mobile phones, PCs, gaming consoles, and smart televisions, and they access the web from a wider variety of locations than Yakuts, according to a representative national sample of 765 internet users. The characteristics of Generation Z are largely unclear because there has not been much research.

### Goal of This Study

To further support the emphasis of this study, empowering Generation Z with digital health literacy skills can lead to better health outcomes and a more informed population. Therefore, this analysis compares health information behavior between Generation Z and older generations. This study explored the relationship between information inquiries with digital health literacy and health empowerment. By doing so, we can better understand how to equip the next generation with the tools and useful information necessary to make informed decisions regarding their health and well-being in the digital age.

### Research Questions

#### HISB Overall

HISB is the term used to describe looking for information about health, dangers to it, illnesses, and ways to safeguard it [16,17]. The methods used by different generations to utilize information sources vary [18,19]. Traditional media sources, such as television, radio, newspapers, and social media as well as professional channels (such as medical professionals and health care practitioners) and layperson communication (such as with family members) can be used to gather information [20,21].

With their interest in health information and the gradual adoption of social media by older people, older Chinese adults have started using WeChat to access health information. For example, a cross-sectional survey was conducted in China to examine the specific patterns and motivating factors that influence how older adults obtain health information [22]. According to their 336 self-reported findings, there are 3 types of behavior (actively seeking, passively browsing, and long-term collecting) when it comes to learning how older adults access health information. In addition, emphasis was placed on the connection among various types of health acquisition behavior, health literacy components, and digital literacy.

The internet has increasingly been acknowledged as a key source of health information; thus, an increasing number of Americans report using the internet to obtain health-related information [23-25]. One in every 3 individuals in the United States has reported using the internet to diagnose or learn about a health condition [26]. A similar trend was observed in other populations in China and in European countries [27,28]. As a result, the corresponding research question (RQ) is as follows:

- RQ1: How do HISBs in different communication channels differ between Generation Z and older populations?

#### Digital Health Literacy and HISB

Health literacy is defined as the cognitive and social skills that influence people’s motivation and capacity to acquire, interpret, and implement knowledge in ways that promote and preserve good health [29]. Poor health results and higher morbidity and mortality rates are associated with low health literacy [30]. People with poor health literacy are more likely to have trouble using and accessing health care facilities, which is a major factor in health inequalities [31]. It has been established that having a solid foundation in health literacy is crucial when looking for medical information. People with poor health literacy seek less health information, rely more on alternative information sources, and have a more challenging time understanding medicine labels or health messaging, according to prior research [32].

Health literacy and digital health literacy are 2 distinct but related concepts. Norman and Skinner [33] discussed the differences between them by introducing the concept of eHealth literacy, which refers to the ability to seek, find, understand, and evaluate health information from electronic sources. The authors argue that digital health literacy goes beyond traditional health literacy by encompassing skills such as navigating web-based health information and using digital tools to manage one’s health [34]. People with greater digital health literacy are more likely to manage and access health information from various information sources efficiently and make wise health decisions [35]. In contrast, people with low digital health literacy may need help obtaining and using health information through various communication channels, which could prevent them from accessing crucial health information and services.

Schulz and Nakamoto [36] proposed the need to distinguish between health literacy and patient empowerment by arguing that digital health literacy can facilitate patient empowerment by providing patients with the tools and information they need to play an active role in their health management. Paige et al [37] examined the differences in digital health literacy between rural and urban adults in the United States. The authors found that rural adults had lower levels of digital health literacy than urban adults, which could have affected their ability to access and use web-based health information. Chang and Schulz [38] sought to develop a comprehensive measurement tool to assess Chinese patients’ eHealth literacy regarding chronic diseases. This study was based on the eHealth Literacy Scale and adapted to the Chinese context by analyzing 347 patients in hospitals. Therefore, 4 factors related to digital health literacy were identified: information and skill, cognitive and affective attitudes, web-based communication, and self-efficacy.

It is simple to predict a favorable relationship between internet use and digital health literacy. However, it is essential to consider how Generation Z and older groups selected different HISB channels and how these differences led to increased digital health literacy. It is logical that members of Generation Z, who have grown up with more exposure to and experience with digital technologies, would demonstrate greater levels of digital literacy. Generation Z members are among the most prominent users of digital health services and technology compared to earlier generations, despite being frequently ignored in scholarly studies.

The relationship between digital health literacy and different channels of informational communication about health has not received much research attention. For example, a study involving 522 Hungarians found that Generation Z had higher levels of digital health literacy than did earlier generations. Although some studies have not directly compared Generation Z with earlier generations, age has shown strong links with digital health literacy [5]. Consequently, we chose the following 2 study questions regarding digital health literacy:

- RQ2: How does digital health literacy differ between Generation Z and older populations?
- RQ3: What is the relationship between digital health literacy and HISB in different communication channels?

#### Health Empowerment and HISB

Health empowerment emphasizes one’s awareness of one’s capacity to participate successfully in decisions regarding health and health care [39]. Improved disease management and patient outcomes can be attributed to greater patient involvement [40-42]. Previous studies have indicated that feeling empowered and seeking health information are related [4,5]; thus, one of the key strategies for empowering people is active information seeking, so that people are more capable of safeguarding themselves and having fruitful interactions with medical professionals.

Although researching web-based health issues can result in more proactive disease management and decision-making, the use of web-based false material has drawn considerable attention [43]. For instance, misinformation or disinformation regarding COVID-19’s causes, transmission, and treatments may have prevented some people from implementing effective preventive measures during the pandemic [44]. However, medical professionals can give individual personalized and accurate health information to consumers.

Few studies have explored in detail the relationship between health empowerment and learning about it from multiple sources in an effort to address this research gap. Given the exceptional caliber of health information provided on the web, it is critical to ascertain whether health empowerment differs between Generation Z and older groups. Therefore, we have added the following question:

- RQ4: How do Generation Z and older populations differ in terms of health empowerment?

Health empowerment and HISB are closely related, as individuals who feel empowered in their health are more likely to engage in proactive HISB to make informed decisions about their health. Previous research has shown that different communication channels can affect the relationship between health empowerment and HISB. For example, a study by Chang and Schulz [38] in 2018 found that individuals who reported high levels of health empowerment were more likely to engage in HISB via web-based channels such as websites and social media.

In comparison, traditional communication channels such as face-to-face interactions with health care professionals may have a more direct impact on health empowerment. In 2017, Sak et al [45] investigated the predictive power of psychological empowerment and health literacy on older patients’ participation in health care. This cross-sectional population-based study was conducted using a sample of 826 older adults in Switzerland. The results showed that both psychological empowerment and health literacy were positively associated with older patients’ participation in health care, with psychological empowerment having a more substantial impact. The authors suggest that health care professionals can play a critical role in promoting health empowerment and HISB among their patients. The relationship between health empowerment and HISB can vary depending on the communication channels used. Understanding the impact of different channels on this relationship is essential for developing effective interventions to promote health empowerment and HISB in diverse populations. Thus, we elaborate RQ 5 as follows:

- RQ5: What is the relationship between HISB and health empowerment on various communication platforms, especially among members of Generation Z?

## Methods

### Overview

The Health Information National Trends Survey (HINTS) is a national survey that examines how the public perceives and uses health information. The poll was created in accordance with various HINTS projects that were already in place in the United States, conducted by the National Cancer Institute [46]. A HINTS study was adopted by Stoddard and Augustson [47] in 2006 to explore the relationship between health information seeking and lung cancer prevention behaviors among adult smokers in the United States. Their study found that internet-connected smokers were more likely to engage in lung cancer prevention behaviors such as screening and healthy lifestyle choices.

Prior HINTS projects for the Chinese population were conducted in 2012 and 2017. Thus, HINTS has been applied to various studies on cigarette smoking, cancer, food fraud, and HISB in the Chinese context. For instance, in 2022 Chang et al [48] used the HINTS to investigate individuals’ preferences for health information channels and sources of health rumors among Chinese adults in mainland China. The study found that respondents preferred to visit their physicians for health information, whereas they primarily received health rumors through the internet, family members, and friends. These recent studies demonstrate the continued relevance and utility of the HINTS tool in examining public HISBs and related factors.

The present studies used updated questionnaire items and the same basic questions from HINTS-China and HINTS-US. These metrics included the frequency with which people looked for health information from various sources and how much they trusted those sources. Additional questions were added, some of which focused on health empowerment and digital health literacy. As a result, the validated questionnaire questions were divided into 6 categories: social networks, health information, health condition and behavior, health care experience, and HISB.

### Procedure

The first web-based questionnaire survey was conducted in Macao, a Special Administrative Region in China, with the participation of adults on campus who had internet access and university students. WeChat and Macao’s Facebook platforms were used in the pilot study to gauge the efficacy of the questionnaire. To reach as many potential respondents as possible, a Chinese poll was made available on popular regional social media sites such as WeChat, QQ, Tencent Weibo, and Sina Weibo. Users of various social media platforms in China were invited to participate in this cross-sectional web-based study between September and November 2022. The study used the snowball sampling technique.

The screening criteria for respondents included being Chinese, aged ≥18 years, gender, and living in one of the abovementioned regions. The response rate was 76.8%, with 3389 people expressing interest in participating.

### Ethical Considerations

Participation was voluntary and anonymous, and written consent was obtained from all respondents. The questionnaire took approximately 15 to 25 minutes to complete. This cooperative project was supported by the University of Macau in China and the University of Lugano in Switzerland. This study was approved by the Ethics Committee for the Social Sciences and Humanities Research at the University of Macau (SSHRE22-APP093-FSS).

### Measurements

#### HISB measures

Participants were asked about their sources of health information and frequency of seeking health information. Nine information sources of 3 traditional media, including television, radio, and newspapers or magazines; 3 web-based media, including websites or search engines, social media, and health-related or news applications; and 3 interpersonal sources, including physicians or health specialists, family members, and friends or colleagues were measured [48]. Four-point frequency scales were used, ranging from 1 (never) to 4 (always).

#### Digital Health Literacy

Participants were assessed for their ability to navigate digital health information, use health apps and devices, and understand health-related terminology. It was measured using the eHealth Literacy Scale with 8 items designed to assess an individual’s ability to find and evaluate web-based health information [38,49]. Five-point scales were used. The average of the responses was used (Cronbach α=.927), and higher scores indicate a greater level of digital literacy.

#### Health Empowerment

Participants were asked about their level of involvement in health care decision-making, self-management of health conditions, and perceived level of control over their health. Health empowerment was estimated using the Psychological Health Empowerment Scale [50,51]. It contains 8 items assessing an individual’s perceived ability to control their health and understand their illness and their enthusiasm for managing their health. Five-point scales were applied, and a higher score indicates better health empowerment. The average value of the responses was applied (Cronbach α=.894).

#### Health Conditions and Sociodemographic Information

Age was expressed in years, education was defined as the highest grade attained (primary school and below=1; bachelor’s degree and above=6), and gender was expressed as dummy variables, with “female” coded as 0. Retirement, enrollment in school, and unemployment were all classified as 0, whereas employment was coded as 1. A 5-point scale was used to categorize personal monthly income, ranging from less than 1500 Chinese yuan (less than US $205.1; score=1) to less than 20,000 Chinese yuan (less than US $2734.2; score=8). Respondents were scored 0 if they had a chronic illness and 1 if they did not have a chronic illness. A 5-point scale from very unhealthy (=1) to very healthy (=5) was used to gauge general health state. The Patient Health Questionnaire-4, which includes 2 items for depression and anxiety, was used to assess mental health [52]. Higher values suggest a worsening of mental health, according to the average score of these 4 items on a 4-point scale. Information on the survey’s language, measurement, and validity can be found in Multimedia Appendix 1.

### Statistical Analysis

Statistical tests were conducted using SPSS Statistics (version 28; IBM Corp). Two-tailed *t* tests were used to compare the information sources used for HISB, digital health literacy, and health empowerment between Generation Z and older groups. The link among HISB, digital health literacy, and health empowerment was investigated using a multiple regression analysis. Specifically, multiple linear regression was used with eHealth literacy as the dependent variable to examine the connection between HISB and digital health literacy. The association between HISB and health empowerment was examined using multiple linear regression, with health empowerment as the dependent variable. To account for confounding factors, sociodemographic information and medical issues were added as covariates in the regression models. While examining eHealth literacy, health empowerment was also considered as a controlled variable and vice versa. The statistical significance was established at a 95% confidence level.

An exploratory factor analysis was conducted on 9 HISB variables with orthogonal rotation (varimax). The eigenvalues for each factor in the data were obtained, and 3 factors had eigenvalues over the Kaiser criterion of 1 and, in combination, explained 65.783% of the variance. The items clustered on the same factor, suggesting 3 factors representing HISB from the internet (Cronbach α=.70), traditional media (Cronbach α=.79), and interpersonal channels (Cronbach α=.697; Multimedia Appendix 2).

## Results

### Overview

A total of 3387 respondents’ responses resulted in 1862 valid responses that answered every question. The participants, who were divided into 2 groups depending on their ages, Generation Z (aged between 18 and 26 years, n=995) and the older population (≥26 years, n=867), had an average age of 32 (SD 13.58) years.

In total, 64.29% (1197/1862) of the respondents were women, whereas 35.71% (665/1862) were men. Most participants had a bachelor’s degree or higher (1250/1829, 68.34%). In total, 21.79% (396/1817) earned 1500 Chinese yuan or less (US $205.1 or less) per month. Very close to half of the respondents (894/1853, 48.25%) were unemployed in 2022. In terms of health, 23.63% (440/1862) of the participants had a chronic illness, whereas most respondents reported good overall health (mean 2.97, SD 0.804) and moderate mental health (mean 1.78, SD 0.661). Table 1 presents the sociodemographic characteristics of the sample of Chinese respondents in the study.

**Table 1:** Sociodemographic characteristics of the Chinese respondents in 2022 (n=1862).

Variables		Respondents, n (%)
**Gender**		
	Women	1197 (64.29)
	Men	665 (35.71)
**Education (n=1829)**		
	Primary school and below	24 (1.31)
	Junior middle school	58 (3.17)
	High school	239 (13.07)
	Junior college	258 (14.11)
	Bachelor’s degree	982 (53.69)
	Higher bachelor’s degree	268 (14.65)
**Monthly income, Chinese yuan (US $) (n=1817)**		
	≤1500 (≤205.1)	396 (21.79)
	1501-2000 (205.2-273.4)	96 (5.28)
	2001-3000 (273.6-410.1)	152 (8.37)
	3001-5000 (410.3-683.5)	329 (18.11)
	5001-8000 (683.7-1093.7)	255 (14.03)
	8001-12,000 (1093.8-1640.5)	178 (9.8)
	12,001-20,000 (1640.6-2734.2)	197 (10.84)
	>20,000 (>2734.2)	214 (11.78)
**Employment (n=1853)**		
	Unemployed	894 (48.25)
	Employed	959 (51.75)
**Chronic diseases**		
	No	1422 (76.37)
	Yes	440 (23.63)

### HISB Results

Overall, interpersonal channels were the most common source of health information (mean 2.68, SD 0.673), followed by the internet (mean 2.67, SD 0.601), and traditional media (mean 2.18, SD 0.728). Older generations were generally more likely to look for health information than Generation Z (t_1861_=6.176; *P*<.001); the older group was more likely to use traditional media (t_1861_=10.541; *P*<.001) and interpersonal channels (t_1861_=5.569; *P*<.001). This finding relates to RQ1, which addresses HISB.

In contrast to the older generation, Generation Z was more likely to find health-related material on the web (t_1861_=−2.022; *P*=.04). In particular, Generation Z was more likely to use social media (t_1861_=−3.009; *P*=.003) and websites or search engines (t_1861_=−2.933; *P*=.003) to identify health issues. Compared with older people, who tend to use a variety of information sources, including traditional media and interpersonal sources, younger people rely more heavily on the internet. Using the 2-tailed *t* test to address HISB, Table 2 evaluates various health information sources by Generation Z and the older population.

**Table 2:** A 2-tailed *t* test comparison of different sources of health information for Chinese Generation Z and older generations (n=1862).

Variables		Generation Z (n=995), mean (SD)	Older population (n=867), mean (SD)	*t* test (*df*)	*P* value
**Health information channels**					
	Overall health information seeking^a^	2.45 (0.497)	2.6 (0.521)	6.176 (1861)	<.001
	Interpersonal sources^b^	2.63 (0.605)	2.79 (0.586)	5.569 (1861)	<.001
	Traditional media^c^	2.02 (0.694)	2.37 (0.723)	10.541 (1861)	<.001
	The internet^d^	2.71 (0.664)	2.65 (0.682)	−2.022 (1861)	.04
**Specific channels**					
	Physician or health specialist	2.56 (0.778)	2.81 (0.759)	7.162 (1861)	<.001
	Family member	2.78 (0.823)	2.8 (0.727)	0.568 (1861)	.57
	Friend or colleague	2.56 (0.758)	2.74 (0.674)	5.633 (1861)	<.001
	Television	2.33 (0.846)	2.57 (0.827)	6.162 (1861)	<.001
	Radio	1.84 (0.839)	2.21 (0.885)	9.176 (1861)	<.001
	Magazine or newspaper	1.9 (0.825)	2.34 (0.869)	10.938 (1861)	<.001
	Websites or search engines	2.99 (0.825)	2.88 (0.828)	−2.933 (1861)	.003
	Health-related applications	2.48 (0.872)	2.52 (0.857)	1.079 (1861)	.28
	Social media: WeChat, Weibo, QQ, blogs, or forums	2.65 (0.845)	2.53 (0.876)	−3.009 (1861)	.003

^a^The average score for seeking health information across all 9 channels.

^b^The average score for seeking health information from a physician or health specialist, family member, and friend or colleague.

^c^The average score for seeking health information from television, radio, magazine, or newspaper.

^d^The average score for seeking health information from websites or search engines, health-related or news applications, and social media (WeChat, Weibo, QQ, blogs or forums).

### Digital Health Literacy and HISB

In response to RQ2, 2 groups of respondents (Generation Z vs the prior generation) stated that they had comparable levels of digital health literacy. High levels of digital health literacy were indicated by the respondents (mean 3.47, SD 0.692), although there was no discernible difference between the levels of the 2 groups (t_1861_=−1.449; *P*=.15). Digital health literacy was substantially predicted by psychological discomfort, empowerment with regard to one’s health, and internet search activity for health information. Particularly, it was discovered that psychological anguish (Generation Z: β=.094, *P*=.002; older population: β=.075, *P*=.04), health empowerment (Generation Z: β=.526, *P*<.001; older population: β=.438, *P*<.001), and HISB via the internet (Generation Z: β=.192, *P*<.001; older population: β=.337, *P*<.001) were favorably correlated in 2 age groups.

For Generation Z, the age factor is a significant predictor of digital health literacy (β=−.035; *P<*.001), and interpersonal networks was also a significant predictor of digital literacy (β=.096; *P=*.008). It was not surprising to see that accessing the internet as a source of health information is the strongest predictor of digital health literacy, according to RQ3. Table 3 presents the results of the multiple linear models for digital health literacy in relation to HISB.

**Table 3:** Multiple linear regression for digital health literacy of the health information–seeking behavior through communication channels.

Variables		Generation Z (n=995; model 1a)^a^		Older population (n=867; model 1b)^b^	
		Standardized coefficient (95% CI)	*P* value	Standardized coefficient (95% CI)	*P* value
Age (years)		−0.035 (−0.055 to −0.015)	<.001	−0.004 (−0.008 to 0.000)	.08
Gender		0.022 (−0.059 to 0.102)	.60	0.076 (−0.009 to 0.160)	.08
Education		0.029 (−0.014 to 0.073)	.19	0.010 (−0.029 to 0.050)	.60
Monthly income (Chinese yuan)		0.015 (−0.005 to 0.035)	.15	0.017 (−0.006 to 0.040)	.15
Occupation		0.101 (−0.007 to 0.209)	.07	0.063 (−0.048 to 0.173)	.27
Chronic disease		−0.022 (−0.123 to 0.079)	.67	−0.034 (−0.127 to 0.059)	.47
Self-rated health		−0.034 (−0.082 to 0.014)	.16	−0.003 (−0.054 to 0.061)	.91
Psychological distress		0.094 (0.035 to 0.154)	.002	0.075 (0.004 to 0.146)	.04
Health empowerment		0.526 (0.460 to 0.593)	<.001	0.438 *(*0.352 to 0.523)	<.001
**Health information seeking**					
	The internet	0.192 (0.127 to 0.256)	<.001	0.337 (0.267 to 0.406)	<.001
	Traditional media	0.011 (−0.047 to 0.070)	.70	0.034 (−0.031 to 0.099)	.31
	Interpersonal sources	0.096 (0.026 to 0.167)	.008	0.025 (−0.050 to 0.100)	.51

^a^*R*^2^=0.323.

^b^*R*^2^=0.304.

### Health Empowerment and HISB

The older group had considerably more empowerment regarding health (t_1861_=5.194; *P*<.001). Therefore, the findings address RQ4, indicating that Generation Z (mean 3.70, SD 0.634) may have less control over how they manage their health than their counterparts (mean 3.84, SD 0.500).

Respondents reported high levels of health empowerment (mean 3.76, *SD* 0.579).

Table 4 presents a number of investigations that demonstrated the following: self-rated health was poorer for younger people but better for older adults (Generation Z: β=−.169; *P*<.001; older population: β=.137; *P*<.001; see model 2a and 3a), whereas psychological distress benefits the young but harms older adults (Generation Z: β=.109; *P*<.001; older population: β=−.156; *P*<.001; see model 2a and 3a). Another meaningful predictor of health empowerment in both generations was the inquiries for health information from interpersonal sources (Generation Z: β=.107; *P*<.001; older population: β=.066; *P*=.02; model 2a and 3a).

Physicians, family members, and friends or coworkers were entered separately into regression models for better comprehension. The most effective interpersonal channel for predicting increased health empowerment was talking to physicians (Generation Z: β=.070; *P*=.002; older population: β=.089; *P*<.001; model 2b and 3b). At the same time, there was no association between HISB and empowerment of family members (Generation Z: β=.036; *P*=.12; older population: β=−.015; *P*=.57) and friends or colleagues (Generation Z: β=−.002; *P*=.94; Older population: β=−.014; *P*=.63). The only significant and promising predictor of health empowerment was the response to RQ5 regarding consulting physicians for health-related information. Table 4 shows the regression outcomes for health empowerment.

**Table 4:** Multiple linear regression for the health empowerment of health information–seeking behavior through communication channels^a^.

Variables		Generation Z (n=995)						Older population (n=867)				
		Model 2a^b^			Model 2b^c^			Model 3a^d^			Model 3b^e^	
		Standardized coefficients (95% CI)	*P* value	Standardized coefficients (95% CI)		*P* value	Standardized coefficients (95% CI		*P* value	Standardized coefficients (95% CI		*P* value
Age (years)		0.021 (0.004 to 0.038)	.02	0.021 (0.004 to 0.039)		.02	0.001 (−.002 to 0.004)		.48	0.001 (−0.002 to 0.004)		.50
Gender		0.042 (−0.027 to 0.110)	.23	0.040 (−0.028 to 0.109)		.25	−0.086 (−0.149 to −0.023)		.008	−0.079 (−0.142 to −0.016)		.01
Education		0.050 (0.013 to 0.088)	.008	0.048 (0.011 to 0.085)		.01	0.019 (−0.010 to 0.048)		.20	0.017 (−0.012 to 0.047)		.25
Monthly income (Chinese yuan)		−0.007 (−0.024 to 0.010)	.41	−0.006 (−0.023 to 0.011)		.496	−0.028 (−0.045 to −0.010)		.002	−0.028 (−0.045 to −0.010)		.002
Occupation		−0.039 (−0.132 to 0.053)	.41	−0.042 (−0.135 to 0.050)		.37	0.053 (−0.029 to 0.136)		.21	0.056 (−0.027 to 0.138)		.19
Chronic disease		0.193 (0.108 to 0.278)	<.001	0.199 (0.113 to 0.284)		<.001	0.051 (−0.018 to 0.121)		.15	0.058 (−0.011 to 0.128)		.100
Self-rated general health		−0.169 (0.130 to 0.209)	<.001	0.171 (0.131 to 0.210)		<.001	0.137 (0.096 to 0.179)		<.001	0.138 (0.096 to 0.179)		<.001
Psychological distress		0.109 (−0.159 to −0.059)	<.001	−0.107 (−0.157 to −0.057)		<.001	−0.156 (−0.208 to −0.104)		<.001	−0.151 (−0.203 to −0.099)		<.001
Digital health literacy		0.383 (0.334 to 0.431)	<.001	0.379 (0.330 to 0.428)		<.001	0.244 (0.197 to 0.292)		<.001	0.239 (0.191 to 0.287)		<.001
**Health information seeking**												
The internet		−0.015 (−0.071 to 0.041)	.60	−0.009 (−0.065 to 0.047)		.75	−0.008 (−0.063 to 0.047)		.78	−0.007 (−0.062 to 0.048)		.80
Traditional media		0.049 (−0.002 to 0.099)	.06	0.056 (0.005 to 0.106)		.03	0.040 (−0.008 to 0.088)		.10	0.046 (−0.003 to 0.094)		.06
**Interpersonal sources**		0.107 (0.047 to 0.167)	<.001				0.066 (0.010 to 0.122)		.02			
	Physician or health specialist	N/A^f^		0.070 (0.025 to 0.115)		.002	N/A			0.089 (0.043 to 0.135)		<.001
	Family member	N/A		0.036 (−0.009 to 0.081)		.12	N/A			−0.015 (−0.066 to 0.036)		.57
	Friend or colleague	N/A		−0.002 (−0.053 to 0.049)		.94	N/A			−0.014 (−0.068 to 0.041)		.63

^a^Health information seeking from physicians or specialists, family members, and friends or colleagues was entered into the regression as 3 variables. Their average value, which was used to denote health information–seeking behavior in interpersonal sources, was not included in the model.

^b^*R*^2^=0.382.

^c^*R*^2^=0.384.

^d^*R*^2^=0.274.

^e^*R*^2^=0.283.

^f^N/A: not applicable.

## Discussion

### Principal Findings

Analysis of the data gathered from various Chinese communication platforms revealed a strong and favorable correlation between HISB and health empowerment among Generation Z. However, the ability of people to take charge of their own health continues to be significantly affected by physicians’ interactions with one another. Table 4 presents the results of several noteworthy investigations that demonstrate the following: while self-rated health is worse for younger people but better for older people, psychological discomfort benefits young people but damages older people. During the COVID-19 pandemic, Generation Z has observed a loss in self-rated health, which is a sign of underlying physical and mental diseases that are frequently challenging to diagnose using conventional health markers such as body weight and height.

Social media platforms such as WeChat, Weibo, and Douyin were found to be the most popular platforms for seeking health information among Generation Z in China. Our study found that older people engage in more social interactions, use traditional media, and have greater control over their health, despite using the internet substantially less than Generation Z. Increased digital health literacy is positively connected with higher use of health information. To further improve Generation Z’s health and well-being, additional research is needed to define the causal linkages among health information seeking, digital health literacy, and health empowerment.

A crucial topic in promoting understanding of health and wellness problems is that members of Generation Z have stated that they are at ease searching for health information on various platforms such as apps and websites. For instance, Ping An Good Doctor, a health care platform in mainland China with more than 12,000 health care service providers and almost 23 million daily active users, was the most widely used medical app in China as of December 2022 [53]. The mobile portal offers web-based appointment scheduling, real-time medical consultations, and a conversation board for health-related topics. These top medical apps have become increasingly popular in China by reducing hospital wait times and fostering better patient-physician contact.

There is no direct connection between Generation Z’s desire for health empowerment and the use of media, including traditional and internet sources. The only source of health information acquired associated with empowerment is from physicians or health care experts. This finding suggests that simply seeking health information on the web and through family members or friends may not empower individuals to take control of their health. Instead, advice and guidance from physicians are necessary for individuals to feel empowered.

There are numerous reasons for this finding. It makes sense to assume that people who can manage their own health have the propensity to interact with medical professionals and seek their advice, actively fulfilling the theoretical prediction [45,54]. Another explanation is that patients who receive health information from physicians more frequently are informed by the information provided by experts, which fosters their interest or perception of control over their health [39,42,45,49].

Those who looked for health information in these web-based venues also showed higher levels of health empowerment. These results imply that HISB can play a significant role in fostering health empowerment, especially among Generation Z, and that it should be seriously considered when developing health communication strategies for this demographic. However, the significance of the role physicians play in promoting patient empowerment has been stressed in motivating patients in both Chinese age groups. The findings should help health care professionals and policymakers develop targeted health promotion messaging in campaigns that consider the various preferences and habits of both age groups. In addition, it aids decision-makers in distinguishing between socioeconomic factors and media use and information consumption, particularly in challenging situations.

### Comparison With Prior Work

According to our analysis, Generation Z in China is predicted to share the views and aspirations of prior generations [22]. It is not surprising that, in line with earlier research [55,56], internet sources constitute Generation Z’s primary source of information on health and well-being, surpassing traditional media and physician visits.

When gaining information about their health from a variety of sources, the younger Chinese generations respond in different ways. The younger generation tends to seek out health information less frequently than the older generation, despite using search engines and social media sites more frequently. Although the 2 populations used the internet to varying degrees, they had comparable levels of digital health literacy, which is consistent with the subject. However, this outcome contradicts earlier research by Lee et al [7] in 2015, which claimed that older people have lower levels of digital health literacy. The process used to gather the data may help describe it because older people who meet and respond to the survey may have superior internet use skills than their peers. However, the results argued that older people showed greater health empowerment than Generation Z, which is consistent with earlier research [5], indicating that older generations believed they were more competent in managing their health and more active.

Age has shown strong correlations with digital health literacy within the Generation Z group, partially supporting the findings of a prior study [5]. Contrary to earlier studies [37,57], older individuals demonstrated stronger levels of digital health literacy and greater confidence in their web-based information-seeking and interaction skills.

### Limitations

The current research has several shortcomings, each of which offers the possibility for further investigation. Psychological factors may be particularly significant in information demands, prior contact with health care professionals, and the conceptualization of HISB, according to earlier studies [45,51,58]. Future studies could attempt to use a longer and more varied list of explanatory variables to enhance the amount of variance that can be explained. The relative importance of various elements in explaining variations in HISB should be the main topic of future research. Politicians can use this information to set optimal priorities and use their limited resources more effectively.

We were unable to determine whether the information sources were reliable in terms of cause and effect. Future academics should consider this normative investigation as a crucial line of inquiry, which we leave unexplored. Another restriction was the snowballing technique used to generate responses via social media messages, internet advertisements, and researchers’ networks. Only 15.47% of the Chinese adults had a junior college degree or higher, according to the national census statistics from 2020 [59], which shows that our participants were more likely to be female and better educated than the census’s assumption. In addition, even though they comprised 48.8% of the population, there were more women than men in our sample. The last but not least drawback was that using questionnaire surveys will inevitably be susceptible to response bias, where respondents might skip questions. This could lead to a large amount of incomplete data and reduce the validity of the survey findings.

### Conclusions

The survey revealed that younger generations, notably Generation Z, are more inclined to use social media platforms to look up health information on the web. The potential of HISB as a tool for advancing health information and empowerment was also highlighted. While disseminating health information, health practitioners and professionals should consider the preferences of various generations, according to the discovery of unique choices of health information sources across the two age groups.

Overall, empowering Generation Z with digital health literacy skills is crucial for promoting healthy behaviors and ensuring better health outcomes in the future. As digital technology continues to advance, it is essential to equip the next generation with the skills and information necessary to navigate the digital world and make informed decisions about their health. The results of this study contribute to the growing body of information on HISB, digital health literacy, and health empowerment among different age groups. It will also provide recommendations for health care providers and policymakers to improve the health literacy and empowerment of Generation Z and older adults.

In conclusion, the cross-generational analysis of HISB revealed the importance of empowering Generation Z with the necessary skills and information to access, evaluate, and use health information on the web. By comparing the digital health literacy levels of this generation to those of older people, we identified potential health disparities that may arise if younger generations lack these critical skills. The analysis also highlights the impact of performance expectancy, facilitating circumstances, and digital health literacy on behavioral intentions, emphasizing the importance of promoting digital health literacy and health empowerment among Generation Z.

## Appendix

Multimedia Appendix 1 Overview of variables.

Multimedia Appendix 2 Exploratory factor analysis of health information–seeking behavior: rotated factor loadings.

## Abbreviations

HINTS: Health Information National Trends Survey
HISB: health information–seeking behavior
RQ: research question

## Data Availability

The data sets generated during or analyzed during this study are available from the corresponding author (AC) upon reasonable request.
