# Supplementary material for: Predicting and Empowering Health for Generation Z by Comparing Health Information Seeking and Digital Health Literacy: Cross-Sectional Questionnaire Study
Source: J Med Internet Res. 2023 Oct 30;25:e47595. doi: 10.2196/47595 (PMC10644182; doi:10.2196/47595)
Supplement: Multimedia Appendix 1 [file jmir_v25i1e47595_app1.docx]

**Multimedia Appendix 1**

Overview of variables.

| Variable | Questionnaire | Scaling details |
| --- | --- | --- |
| Health information seeking behavior | “Have you encountered health or medical information from [information source] in the past 12 months?” | 4-category frequency scale, ranging from never (= 1) to always (= 4) |
| Digital health literacy | “Please indicate the extent to which each statement applies to you personally:”   - - 1. I know how to find helpful health resources on the Internet.     2. I know how to use the Internet to answer my health questions.     3. I know what health resources are available on the Internet.     4. I know where to find helpful health resources on the Internet.     5. I know how to use the health information I find on the Internet to help me.     6. I have the skills to evaluate the health resources I find on the Internet.     7. I can tell high quality from low-quality health resources on the Internet.     8. I feel confident in using information from the Internet to make health decisions. | Averaged scores of 8 items (Cronbach alpha = .927).  5-point scale from strongly disagree = 1 to strongly agree = 5 |
| Health empowerment | “How much do you agree with the following statements?”   1. I will use the necessary means and goods to manage health effectively. 2. I can understand my disease better than anyone. 3. I can motivate myself to manage my health and make a better life. 4. I can make every possible effort to achieve health goals. 5. I am enthusiastic about my efforts to manage my health. 6. I know where I can ask for help to manage my disease. 7. I can manage my disease conditions. 8. I can make a realistic health plan. | 5-point scale from strongly disagree = 1 to strongly agree = 5 |
| Self-rate general health | “In general, how would you say your health?” | 5-point scale from very bad = 1 to very good = 5. |
| Psychological distress | “Over the past 2 weeks, how often have you been bothered by any of the following problems?”   1. Little interest or pleasure in doing things. 2. Feeling down, depressed, or hopeless. 3. Feeling nervous, anxious or on edge. 4. Not being able to stop or control worrying. | Averaged scores of 4 items (Cronbach alpha = .904).  4-point scales from not at all = 1 to nearly every day = 4. |
