# Supplementary material for: Predicting and Empowering Health for Generation Z by Comparing Health Information Seeking and Digital Health Literacy: Cross-Sectional Questionnaire Study
Source: J Med Internet Res. 2023 Oct 30;25:e47595. doi: 10.2196/47595 (PMC10644182; doi:10.2196/47595)
Supplement: Multimedia Appendix 2 [file jmir_v25i1e47595_app2.docx]

**Multimedia Appendix 2**

Exploratory factor analysis of health information-seeking behavior: Rotated factor loadings

| Items^a^ | The internet | Traditional media | Interpersonal channels |
| --- | --- | --- | --- |
| Physician or health specialist |  |  | **.518** |
| Family member |  |  | **.721** |
| Friend or colleague |  |  | **.620** |
| Television |  | **.588** |  |
| Radio |  | **.896** |  |
| Newspaper or magazine |  | **.664** |  |
| Website or search engines | **.534** |  |  |
| Applications: health-related or news applications | **.682** |  |  |
| Social media | **.629** |  |  |
| Eigenvalues | 1.096 | 3.484 | 1.341 |
| % of variance | 12.181 | 38.708 | 14.895 |
| Cronbach alpha | .700 | .792 | .697 |

^a^: Eigenvalues smaller than 0.30 are suppressed; Items with the bold eigenvalue represent the factor which it subordinated to.
